# Association between serum platelet level and dermatitis rash: Results from the China Health and Nutrition Survey

**DOI:** 10.1371/journal.pone.0347031

**Published:** 2026-05-04

**Authors:** Yue Jiang, Ding Li, Xiaojie Ding, Zhan Zhang, Xiaoxuan Ma, Haotian Xu, Guangyuan Cheng, Ying Zhang, Yi Ru, Ying Luo, Wencheng Jiang, Ruiping Wang, Bin Li, Yue Luo, Jiankun Song

**Affiliations:** 1 Department of Dermatology, Yueyang Hospital of Integrated Traditional Chinese and Western Medicine, Shanghai University of Traditional Chinese Medicine, Shanghai, China; 2 Institute of Dermatology, Yueyang Hospital of Integrated Traditional Chinese and Western Medicine, Shanghai University of Traditional Chinese Medicine, Shanghai, China; 3 Shanghai Skin Disease Hospital, Institute of Dermatology, School of Medicine, Tongji University, Shanghai, China; King Fahd Military Medical Complex, SAUDI ARABIA

## Abstract

Dermatitis rash (DR), a prevalent inflammatory skin disorder marked by erythema, pruritus, and scaling, poses diagnostic challenges due to overlapping phenotypes with atopic dermatitis and psoriasis. This study examines the relationship between serum platelet (PLT) levels and DR, as the PLT-DR link remains unknown. We used the China Health and Nutrition Survey (CHNS) database, 7,337 participants (172 DR cases, 7,165 controls) were analyzed. Baseline characteristics were compared via weighted chi-square tests. Multifactor regression models and weighted logistic regression assessed the PLT-DR relationship, while restricted cubic splines (RCS) explored nonlinear associations. The impact of age and sex was also evaluated. We found PLT levels significantly reduced DR risk (model 1: odds ratios (*OR*) = 0.9974 [95% confidence intervals (CI) = 0.9951–0.9998], *P* = 0.0316; model 2: *OR* = 0.9976 [95% CI = 0.9953–0.9999], *P* = 0.0490; model 3: *OR* = 0.9975 [95% CI = 0.9950–0.9999], *P* = 0.0398). Covariates such as previous smoking and rural residency were significantly associated with DR. Nonlinear models and risk stratification confirmed these findings, with female sex moderating the PLT-DR relationship. This elucidates that higher PLT levels may independently lower DR risk, suggesting diagnostic potential. PLT thresholds may enhance risk prediction models, aiding early intervention in high-risk populations.

## 1. Introduction

Dermatitis, characterized by erythema, pruritus and scaling, and rash, presenting as macules or papules, are prevalent inflammatory skin conditions [1]. Epidemiologic surveillance reveals that contact dermatitis affects 15%−20% globally, escalating to 30% in high-risk occupational cohorts [2,3]. Atopic dermatitis (AD) demonstrates striking pediatric predominance, with prevalence rates of 15%−20% in industrialized nations versus 12.9% in China, exhibiting a progressive upward trajectory [4,5]. Concurrently, rash incidence shows parallel escalation, attributed to escalating environmental stressors, shifting lifestyle patterns, and genetic predispositions [6]. Diagnosis of dermatitis rash (DR) remains clinically challenging owing to its overlapping phenotypes. For instance, the substantial similarity between AD and psoriatic lesions contributes to 20%−30% misdiagnosis rates in early-stage evaluations [7]. Emergency department assessments further complicate risk stratification, as benign rashes often mimic life-threatening infectious exanthems [8]. Elucidating the precise risk factors for DR thus holds urgent clinical utility.

Serum platelet (PLT) count, a critical hematological indicator, reflects circulating PLT levels with a normative range of (100–300)×10⁹/L [9]. PLT mediates essential biological functions, including hemostasis, wound healing, and immunoinflammatory regulation, through mechanisms such as inflammatory mediator release, leukocyte recruitment, and vascular repair facilitation [10]. Clinically significant PLT deviations correlate with diverse pathologies: thrombocytopenia may indicate immune thrombocytopenia [11], aplastic anemia [12], acute leukemia, or hypersplenism [13,14], and thrombocytosis is associated with myeloproliferative disorders like essential thrombocythemia [15], chronic myeloid leukemia [16], and polycythemia vera [17]. Notably, AD severity is positively correlated with serum immunoglobulin E (IgE) [18], interferon-γ (IFN-γ) [19], and Th17 cytokine levels [20], suggesting potential PLT-mediated mechanisms in DR progression. These immunoregulatory molecules may modulate PLT activation states, potentially exacerbating cutaneous inflammation through PLT-mediated pathways. Given the plausible intersection between PLT dysfunction and inflammatory dermatoses, systematic investigation into PLT as a biomarker for DR warrants prioritization. Elucidating this relationship could advance pathophysiological understanding and inform novel diagnostic/therapeutic strategies for DR management.

The China Health and Nutrition Survey (CHNS) is a large-scale longitudinal household-based investigation with a multi – purpose methodological framework. It mirrors National Health and Nutrition Examination Surveys (NHANES)‘s standardized biomarker assays and quality control protocols and has extensively probed into the correlations among physiological/clinical parameters, behavioral patterns, and prevalent diseases like cardiovascular pathologies, diabetes mellitus, obesity spectrum disorders, and gastrointestinal conditions across China’s socioeconomic and demographic transitions [21,22]. However, prior CHNS-derived research has largely overlooked the relationship between PLT indices and DR, which is a critical gap in dermatological epidemiology. This study uniquely addresses PLT-dermatitis interactions in China’s transitioning population by leveraging CHNS’s harmonized biomarker protocols and socioeconomic diversity, a focus absent in Western cohorts [23–26].

This investigation leverages the CHNS database to systematically evaluate PLT level-associated risks for DR through four analytical dimensions: comparative baseline profiling between DR patients and healthy controls, risk correlation quantification, stratification analysis, and nonlinear predictive modeling. Furthermore, we elucidate age- and gender-specific modulatory effects on PLT-DR associations. The derived evidence aims to establish an evidence-based framework for optimizing clinical decision-making and therapeutic strategies in DR management.

## 2. Methods

### 2.1. Data collection and participants

The related data to DR were acquired from CHNS database (https://www.cpc.unc.edu/projects/china). This study is a secondary analysis of publicly available data from the CHNS. The original CHNS protocol was approved by the Institutional Review Board of the University of North Carolina at Chapel Hill and the National Institute for Nutrition and Health, Chinese Center for Disease Control and Prevention. Written informed consent was obtained from all participants. After excluding individuals under 18 years of age and those with missing data or no data on exposure factors, covariates, and DR, the 7,337 participants were included in this study from 72,820 participants in 2009 (S1 Fig). The subjects were asked questions: “Rash dermatitis?”. Subjects who answered “yes” served as the DR group, while subjects who answered “no” served as the non-DR group. It is essential to note that the single question, “Rash dermatitis?”, used in the CHNS survey was designed to screen for a wide range of inflammatory skin conditions. Thus, “Dermatitis Rash (DR)” in this study is a broad clinical phenotype based on self-reporting and may encompass a wide range of conditions, including atopic dermatitis, contact dermatitis, psoriasis rash, and other inflammatory rashes of unknown origin. In the absence of more detailed clinical diagnosis, physical examination, or pathologic confirmation information in the database, we were unable to perform further etiologic or phenotypic segmentation of DR.

### 2.2. Definition of variables

The DR was employed as the outcome and PLT level (10^9^/L) was employed as a continuous variable for exposure. Furthermore, some important concomitant variables were also extracted to assess the effect of potential confounders (S1 Table). Weight, 3-day carbohydrate intake, height, 3-day fat intake, body mass index (BMI), 3-day calorie expenditure, and 3-day protein intake were implemented as continuous variables. Age was separated into 3 categories: 18–29 years, 30–59 years, and ≥65 years. Gender was divided into male or female. The nation was divided into Han and other minorities. Smoking status was classified as current smokers, former smokers and never smokers. Residential land was grouped: urban and rural. Education level was categorized into 3 groups: bachelor’s degree or higher, junior high school or lower, high school or vocational high school. Frequency of drinking alcohol was grouped: almost every day, 3–4 times a week, once or twice a week, once or twice a month, no more than once a month, and never. Asthma, diabetes and high blood pressure were grouped: yes or no.

### 2.3. Statistical analysis

All statistical analyses were performed utilizing R software (v 4.2.2). The weighted chi-square test was implemented to analyze the variations in baseline characteristics between non-DR group and DR group (*P* < 0.05). After that, multifactor regression models were built to explore whether PLT level was linked to the risk of DR utilizing “survey” package (v 4.4.1) (http://r-survey.r-forge.r-project.org/survey/) and adjusted OR and 95% CI were computed (*P* < 0.05). Adjustments for covariates were made in the following 3 models: model 1 = unadjusted; model 2 = model 1 + race, gender, and age; model 3 = model 2 + education, 3-day carbohydrate intake, 3-day fat intake, weight, BMI, 3-day protein intake, diabetes, hypertension, place of residence, height, 3-day calorie expenditure, asthma, smoking status, frequency of alcohol consumption. Subsequently, to further determine the association between PLT level and the risk of DR in different subgroups of people, weighted logistic regression analysis was performed based on model 3 and the result was presented utilizing “forestplot” package (v 3.1.1) (*P* < 0.05, Hazard Ratio (*HR*) ≠ 1). Furthermore, to explore the relationship between different PLT levels and DR, the potential nonlinear relationship between the 2 was modeled utilizing RCS (*P* > 0.05). Finally, to explore the differences in the association between PLT and DR risk across various population subgroups, we performed a subgroup analysis. In this analysis, we converted PLT into a binary variable based on the overall median to facilitate the calculation of a consistent effect size and interaction P-value within each subgroup (*P* < 0.05, *HR* ≠ 1) (S2 Fig).

## 3. Results

### 3.1. Baseline characteristics of study subjects

All participants were divided into 2 groups based on whether they had DR. The number of people without DR was 7,165 and with DR was 172 from the baseline characteristics (S2 Table). Of them, smoking and 3-day carbohydrate intake showed significant differences between the two groups, indicating that smoking and 3-day carbohydrate intake were most likely to affect DR (*P* = 0.003). Besides, age (*P* = 0.004), residential land (*P* = 0.004), high blood pressure (*P* = 0.006), 3-day protein intake (*P* = 0.014), and 3-day calorie expenditure (*P* = 0.026) also had significant effects on DR. The P value of PLT was 0.032, indicating PLT level displayed a small but significantly significant negative association with DR. Furthermore, height might also have a relatively weak influence on DR (*P* = 0.046). Although other covariates had no significant effect on DR, they were still considered as common base-adjusted covariates.

### 3.2. Risk correlation and risk stratification in DR

**Fig 1:**
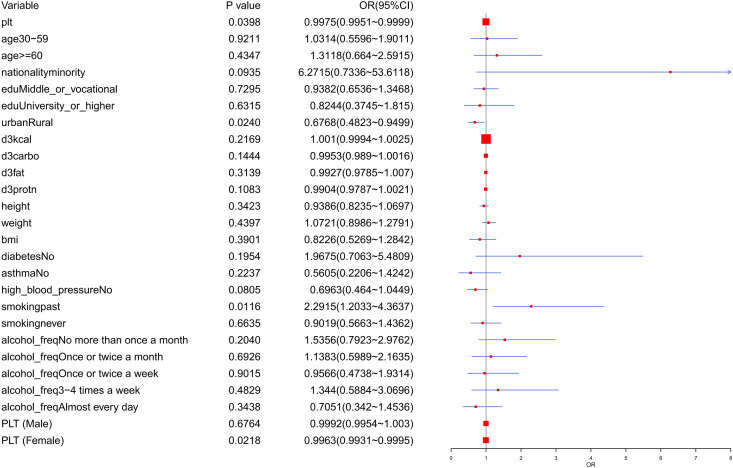
Stratified risk association of PLT and DR. PLT, platelets; BMI, body mass index; kcal, kilocalorie; OR, odds ratios; edu, education.

Based on 3 models, risk correlation analysis indicated the effect of PLT level on DR was not significantly affected by other covariates and PLT was considered to be a weakly protective factor for DR (model 1: *OR* = 0.9974 [95% CI = 0.9951–0.9998], *P* = 0.0316; model 2: *OR* = 0.9976 [95% CI = 0.9953–0.9999], *P* = 0.0490; model 3: *OR* = 0.9975 [95% CI = 0.9950–0.9999], *P* = 0.0398) (Table 1). Risk stratification analysis showed that previous smoking was strongly positively associated with DR risk (*P* < 0.05, *OR* = 2.2915 [95% CI = 1.1660–4.2667]), while rural residency was weakly negatively associated with DR risk (*P* < 0.05, *OR* = 0.6768 [95% CI = 0.4828–0.9517]); they were considered a risk factor and a protective factor for DR, respectively (Fig 1). Besides, there was a significant negative association between PLT level and DR risk, with a lower odds of DR associated with higher PLT level (*P* < 0.05, *OR* = 0.9975 [95% CI = 0.9950–0.9998]). Thus, PLT level was a protective factor for DR.

**Table 1:** Association analysis of participants.

	Group	OR (95% CI)	p_value
model 1	PLT	0.9974(0.9951-0.9997)	0.0316
model 2	PLT	0.9976(0.9953-0.9999)	0.0490
model 3	PLT	0.9975(0.9950-0.9999)	0.0398

Notes: PLT, platelets; OR, odds ratios; CI, confidence intervals.

### 3.3. Nonlinear model between PLT level and DR

**Fig 2:**
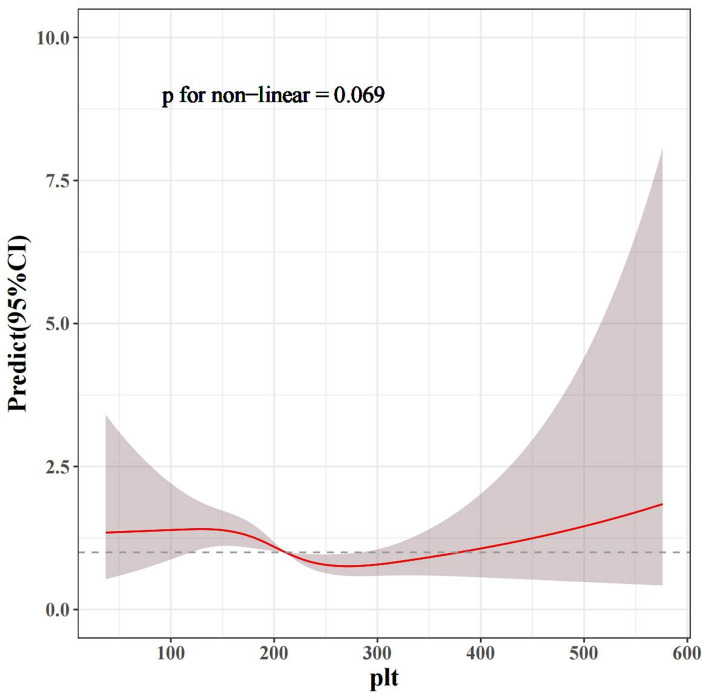
Fitted curve from restricted cubic spline analysis modeling the association between PLT and the DR. The grey band represents the 95% confidence interval. The P value for nonlinearity was 0.069. PLT, platelets; CI, confidence intervals.

A constructed nonlinear model indicated that a potential U-shaped association between PLT level and DR prevalence (*P* = 0.0690). As PLT levels increased, the risk of DR decreased first and then increased (Fig 2).

### 3.4. The risk association between PLT and DR in different subgroups

**Fig 3:**
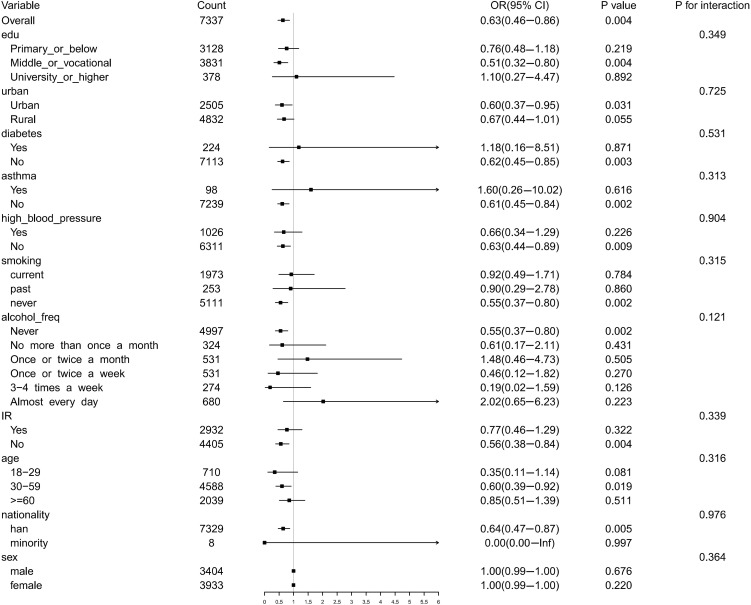
Subgroup analysis forest plot of PLT and DR Risk. The odds ratio (OR) is calculated based on the binary classification PLT. In the figure, “Overall” represents the comprehensive effect of the entire population (n = 7337). Sample size (Count), OR (95% CI), p-value and interaction p-value (P_for_interaction) were labeled for each subgroup. edu, education; freq, frequency; IR, insulin resistance; OR, odds ratios; CI, confidence intervals.

To explore the differences in the risk association between PLT and DR among different population subgroups, we conducted a subgroup analysis. In different subgroups, risk association indicated the effects between PLT and DR in multiple variables were significant, comprising middle/vocational, urban, no diabetes, no asthma, no high blood pressure, never smoke, never drink alcohol, no insulin resistance (IR), people aged 30–59 years, Han ethnicity, and female (*P* < 0.05) (Fig 3). In conclusion, the relationship between PLT and DR was influenced by multiple factors.

## 4. Discussion

DR is an immune-mediated skin disorder characterized by erythema and pruritus, influenced by genetic predisposition, environmental exposures, and aberrant inflammatory pathways [1,6]. The nonlinear analysis suggested a possible U shaped relationship between PLT and DR, although this association did not reach statistical significance (*P* = 0.069) (Fig 2). In the sex stratified analysis, PLT was significantly inversely associated with DR in women (OR: 0.9963, 95% CI: 0.9931 to 0.9995, *P* < 0.05), whereas no significant association was detected in men (Fig 1). These findings indicate that the potential protective effect of higher PLT levels may be confined to females, implying that the role of PLT in the development of dermatitis may be sex dependent. These findings suggest that PLTs may be weak modulators of dermatological inflammation supported by cohort stratification and cubic spline analysis performed on the CHNS database. This effect may also provide insights into mechanistic relationships between hematological parameters and eczema pathogenesis [27,28]. These findings transcend traditional risk models, suggesting that DR pathogenesis may involve the interplay between hematological and immunological factors, as well as sex-dependent mechanisms.

The analysis first revealed a significant difference in smoking status between the DR and non DR groups. Although no previous study has directly reported an association between smoking and DR, existing evidence has shown that smoking is linked to a higher prevalence of atopic dermatitis [29]. This finding may indirectly suggest a potential relationship between smoking and DR. In addition, 3 day carbohydrate intake also differed significantly between the two groups. However, no relevant studies have yet clarified its association with DR, and this issue warrants further investigation. Further analyses indicated that age, place of residence, hypertension, 3 day protein intake, and energy expenditure also showed significant differences between the two groups. Previous studies have suggested that environmental risk factors, including place of residence, diet, and smoke exposure, may influence the development of dermatitis [30]. Age and hypertension have also been reported to be associated with dermatitis [31,32]. Among the variables showing group differences, PLT, as the primary variable of interest, was further included in the association analysis. Although our study identified a statistically significant association between PLT and DR (*P* < 0.05), the effect size was very small (OR = 0.9975). This indicates that, at the individual level, the absolute change in risk associated with a one unit increase in PLT is minimal. Compared with established risk factors such as smoking history (OR = 2.29), the magnitude of the PLT association is substantially weaker, suggesting that a single PLT measurement is unlikely to serve as a strong standalone predictor in clinical practice. Nevertheless, this modest association may still be informative from mechanistic and public health perspectives. First, PLT is a continuous measure with a wide normal range (100–300 × 10⁹/L), and contrasts between individuals at the lower and upper ends of the distribution may translate into more clinically relevant differences in risk. Second, in large scale epidemiological studies, even small but consistently replicated associations can point to previously unrecognized biological pathways or represent meaningful nodes within complex disease networks. Third, PLT is routinely available at low cost, and incorporating it into multivariable prediction models alongside stronger factors, including smoking history and atopic predisposition, may modestly improve overall discrimination.

We observed a possible U shaped pattern between PLT and DR, although the test for nonlinearity was not statistically significant (*P* = 0.069). If DR encompasses heterogeneous inflammatory disorders, such as atopic dermatitis, which is mainly driven by the Th2 pathway, and psoriasis, which is characterized by Th17 related and neutrophil mediated inflammation, platelets may exert distinct effects across these different inflammatory settings. For instance, lower PLT may reduce the release of immunoregulatory platelet derived factors such as TGF beta, thereby aggravating Th2 type inflammation [33]. Conversely, markedly elevated PLT may intensify Th17 and neutrophil related inflammation by strengthening platelet neutrophil interactions or by releasing proinflammatory mediators [34,35]. Such bidirectional effects could jointly shape the observed nonlinear profile. However, because subtype classification data were unavailable in this study, this hypothesis cannot be tested directly. In addition, the wide confidence intervals at the extremes of the curve (PLT < 150 and PLT > 300) indicate that trends in these ranges should be interpreted cautiously, as they may be driven by a small number of extreme observations or measurement variability. Future studies in independent cohorts with clinically confirmed diagnoses should evaluate platelet associations separately for atopic dermatitis, psoriasis, and other specific conditions to clarify the biological heterogeneity underlying this pattern.

Risk stratification analysis identifies former smoking as an independent risk factor for DR (*OR* = 2.2915, *P* < 0.05), its effect size was markedly greater than that of other modifiable factors. Evidence from a Mendelian randomization study indicated that smoking was significantly positively associated with the risk of atopic dermatitis [36], and this finding was further supported by another study [37]. Smoking-induced oxidative stress amplifies the Th2/Th17 imbalance, compromising cutaneous immune tolerance [38]. Notably, tobacco-derived toxins impair PLT-endothelial interactions, potentially negating PLT-mediated protection even in individuals with normal PLT counts [39]. This synergy between smoking and PLT dysfunction suggests that combined interventions—such as smoking cessation and PLT function monitoring—could enhance DR prevention [40]. Public health campaigns targeting smoking behavior may achieve dual benefits for dermatological and cardiovascular health [41,42]. Taken together, these accumulating findings further underscore the importance of incorporating smoking cessation into multidisciplinary strategies for DR prevention.

Subgroup analysis revealed sex-based heterogeneity in the protective role of PLT levels against DR, with a statistically significant effect modification (*P* < 0.05). Subgroup analysis revealed sex based heterogeneity in the association between PLT and DR. Specifically, the inverse association between PLT and DR was significant in females, whereas this relationship appeared weaker and was not statistically significant in males. These findings suggest that the potential protective effect of higher PLT levels may be attenuated in men. Sex hormone related regulation of PLT inflammatory pathways may partly account for this difference [43,44]. Estrogen has been shown to affect platelet activation related markers such as GPVI receptors [45], and genetic variation associated with estrogen receptor signaling may further influence platelet reactivity [46]. These biological differences may help explain why the PLT-DR association was more evident in females in the present study. Nevertheless, because the overall association between PLT and DR was weak, and the subgroup sample size was limited, this interpretation remains preliminary and requires further validation. This sex specific pattern suggests that the clinical interpretation of PLT as a candidate biomarker for DR may need to take sex related differences into account. It is noteworthy that several factors identified in subgroup analyses as potential modifiers of the PLT to DR association, including sex, educational attainment, and place of residence, did not show statistically significant interaction effects in the initial multivariable linear regression models. This discrepancy may reflect methodological differences. Linear models primarily estimate an overall average association between PLT and DR risk, whereas subgroup analyses are more sensitive to heterogeneity within specific populations. For example, the more evident inverse association between PLT and DR among women may be related to estrogen mediated regulation of pathways involved in platelet activation [43–46]. These findings suggest that, when considering PLT as a candidate biomarker for DR, its effect may be contingent on sex and lifestyle factors. Any future clinical use should therefore be interpreted in a stratified manner that accounts for population characteristics.

Smoking was significantly associated with DR, but no causal relationship between the two was established. Although previous studies have reported an association between oxidative stress and dermatitis [47], our data did not provide direct evidence that smoking induced oxidative stress, platelet dysfunction, or altered platelet endothelial interactions are involved in the pathogenesis of DR. Nevertheless, the weak association between platelet count and DR raises the possibility that smoking related oxidative stress may contribute to DR development, with mild platelet involvement potentially acting as a partial mediating factor [48]. In this study, the association between PLT and DR was relatively weak, and this association was mainly observed in women. Platelets may participate in the pathogenesis of DR through mechanisms beyond cell count, such as platelet activation. Future studies may further clarify whether platelet activation is involved in the pathogenesis of skin diseases by directly measuring biomarkers of platelet activation in peripheral blood or in interstitial fluid from lesional and normal tissues. In clinical practice, stratified intervention strategies targeting thrombopoietin or adenosine signaling pathways may be developed by integrating platelet function, hormonal status, and smoking history [49]. Incorporating hematologic mechanisms into the clinical diagnosis and management of skin diseases may further refine risk stratification systems and promote the development of precision medicine in dermatology through biomarker driven strategies, thereby improving therapeutic outcomes [50].

However, this study has certain limitations. First, the causal relationship between DR and PLT remains unclear. Second, in this study, DR was defined using a single self reported question, which lacks diagnostic specificity. Participants responding “yes” may have included individuals with atopic dermatitis, psoriasis, or other forms of dermatitis. In addition, the number of DR cases was limited (n = 172), and some strata in subgroup analyses contained even fewer cases, which may have reduced statistical power. In future work, we will conduct a large scale, multicenter prospective cohort study to longitudinally evaluate whether changes in PLT are causally linked to DR risk. We will also apply standardized diagnostic criteria, incorporating dermatologist assessment and pathology when indicated, to classify DR subtypes more accurately and clarify subtype specific associations between PLT and conditions such as atopic dermatitis and psoriasis. Finally, we will integrate multi omics data with analyses of the cutaneous immune microenvironment to elucidate the molecular mechanisms through which PLT may influence skin inflammation, thereby providing a rationale for targeted interventions.

## 5. Conclusions

Using data from the China Health and Nutrition Survey among Chinese adults, our findings suggest that higher PLT is independently associated with a lower risk of DR, and the relationship may be nonlinear with a possible U shaped pattern. We further observed that sex and smoking history may modify this association. However, given the cross sectional design and the relatively limited number of cases, these results do not establish causality and should be validated in larger, well designed prospective studies. Overall, our study provides preliminary epidemiological evidence for a potential role of PLT in DR. If a causal link is confirmed, monitoring PLT may offer a complementary biomarker perspective for DR risk stratification and clinical assessment.

## Supporting information

S1 Fig Participant Screening Flowchart. (TIF)

S2 Fig Analytical Workflow: Multivariable Modeling Progression. (TIF)

S1 Table Variables and their numbering information table. (DOCX)

S2 Table Baseline characteristics statistical table. (DOCX)
