# Supplementary figures and images for: Association between serum platelet level and dermatitis rash: Results from the China Health and Nutrition Survey
Source: PLoS One. 2026 May 4;21(5):e0347031. doi: 10.1371/journal.pone.0347031 (PMC13138638; doi:10.1371/journal.pone.0347031)

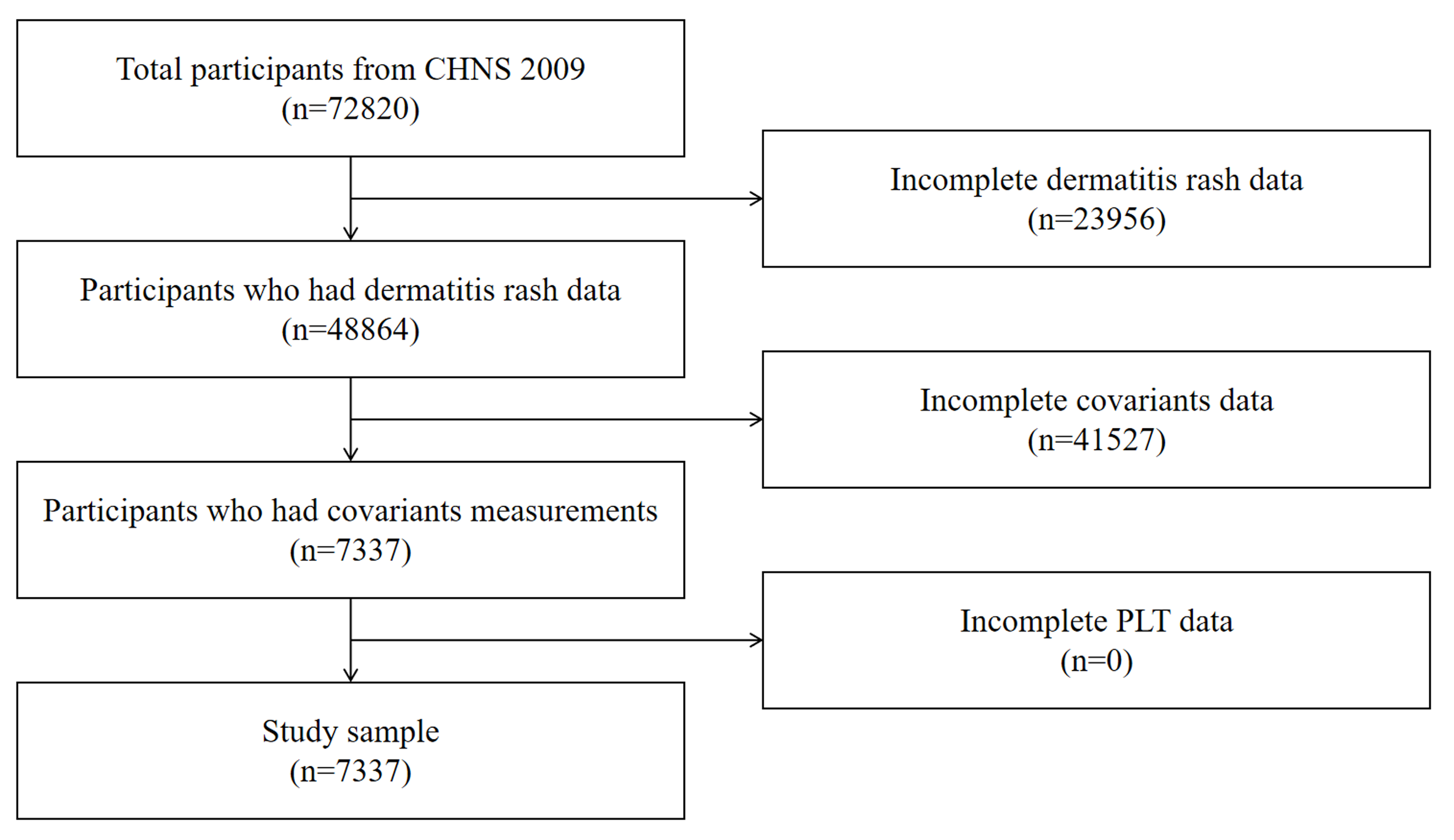

Supplement: S1 Fig — (TIF) [file pone.0347031.s001.tif]

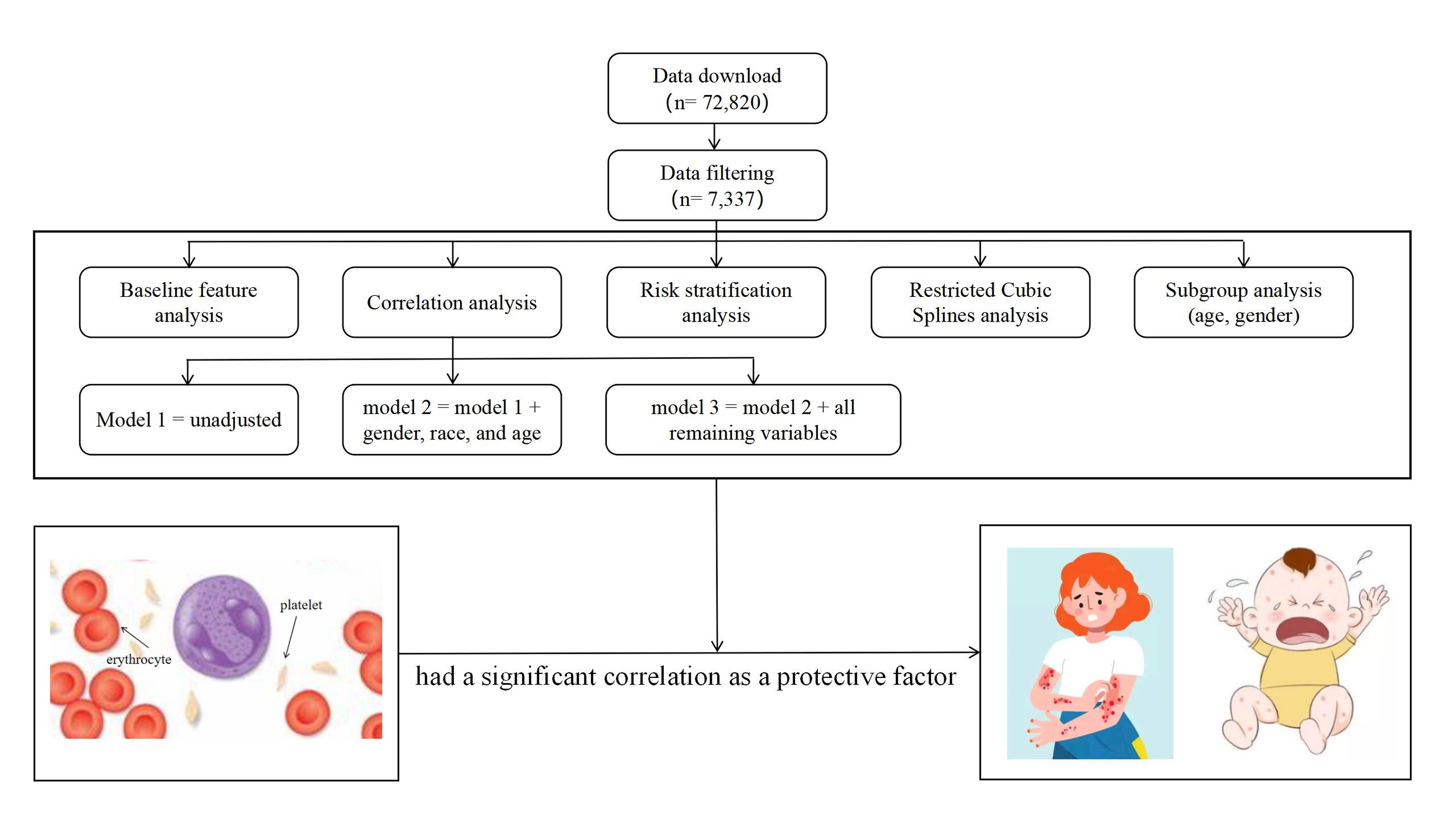

Supplement: S2 Fig — (TIF) [file pone.0347031.s002.tif]
