# Supplementary material for: Association between serum platelet level and dermatitis rash: Results from the China Health and Nutrition Survey
Source: PLoS One. 2026 May 4;21(5):e0347031. doi: 10.1371/journal.pone.0347031 (PMC13138638; doi:10.1371/journal.pone.0347031)
Supplement: S1 Table — (DOCX) [file pone.0347031.s003.docx]

**S1 Table.** Variables and their numbering information table

| variable | Data set and definition number | year |
| --- | --- | --- |
| age | U1_EN | 2009 |
| sex | U1B_EN | 2009 |
| nationality | NATIONALITY | 2009 |
| education | A12 | 2009 |
| urban | T2 | 2009 |
| height | U3_EN | 2009 |
| weight | U2_EN | 2009 |
| bmi | calculated by height andweight | 2009 |
| high_blood_pressure | U22 | 2009 |
| diabetes | U24A | 2009 |
| asthma | U24Q | 2009 |
| d3carbohydrate | D3CARBO | 2009 |
| d3fat | D3FAT | 2009 |
| d3calorie | D3KCAL | 2009 |
| d3protein | D3PROTN | 2009 |
| smoking | U27、U25 | 2009 |
| alcohol_freq | U40 | 2009 |
| PLT | PLT | 2009 |
| dermatitis_rash | M24B_5 | 2009 |
