# Supplementary material for: Association between serum platelet level and dermatitis rash: Results from the China Health and Nutrition Survey
Source: PLoS One. 2026 May 4;21(5):e0347031. doi: 10.1371/journal.pone.0347031 (PMC13138638; doi:10.1371/journal.pone.0347031)
Supplement: S2 Table — (DOCX) [file pone.0347031.s004.docx]

**S2 Table.** Baseline characteristics statistical table

|  | level | No | Yes | p |
| --- | --- | --- | --- | --- |
| n |  | 7165 | 172 |  |
| age (%) | 18-29 | 697 (9.7) | 13 (7.6) | 0.004 |
|  | 30-59 | 4496 (62.7) | 92 (53.5) |  |
|  | >=60 | 1972 (27.5) | 67 (39.0) |  |
| sex (%) | male | 3330 (46.5) | 74 (43.0) | 0.412 |
|  | female | 3835 (53.5) | 98 (57.0) |  |
| nationality (%) | han | 7158 (99.9) | 171 (99.4) | 0.465 |
|  | minority | 7 (0.1) | 1 (0.6) |  |
| edu (%) | Primary_or_below | 3045 (42.5) | 83 (48.3) | 0.320 |
|  | Middle_or_vocational | 3750 (52.3) | 81 (47.1) |  |
|  | University_or_higher | 370 (5.2) | 8 (4.7) |  |
| urban (%) | Urban | 2428 (33.9) | 77 (44.8) | 0.004 |
|  | Rural | 4737 (66.1) | 95 (55.2) |  |
| d3kcal (mean (SD)) |  | 2136.5 (669.5) | 2021.8 (627.3) | 0.026 |
| d3carbo (mean (SD)) |  | 293.7 (102.4) | 270.4 (95.0) | 0.003 |
| d3fat (mean (SD)) |  | 75.2 (41.1) | 74.3 (35.5) | 0.779 |
| d3protn (mean (SD)) |  | 66.3 (23.2) | 61.9 (21.3) | 0.014 |
| height (mean (SD)) |  | 161.2 (8.0) | 160.0 (8.7) | 0.046 |
| weight (mean (SD)) |  | 60.8 (10.1) | 59.5 (11.0) | 0.090 |
| bmi (mean (SD)) |  | 23.3 (3.2) | 23.2 (3.4) | 0.461 |
| diabetes (%) | Yes | 220 (3.1) | 4 (2.3) | 0.736 |
|  | No | 6945 (96.9) | 168 (97.7) |  |
| asthma (%) | Yes | 93 (1.3) | 5 (2.9) | 0.139 |
|  | No | 7072 (98.7) | 167 (97.1) |  |
| high_blood_pressure (%) | Yes | 989 (13.8) | 37 (21.5) | 0.006 |
|  | No | 6176 (86.2) | 135 (78.5) |  |
| smoking (%) | current | 1932 (27.0) | 41 (23.8) | 0.003 |
|  | past | 239 (3.3) | 14 (8.1) |  |
|  | never | 4994 (69.7) | 117 (68.0) |  |
| alcohol_freq (%) | Never | 4878 (68.1) | 119 (69.2) | 0.744 |
|  | No more than  once a month | 313 (4.4) | 11 (6.4) |  |
|  | Once or twice  a month | 519 (7.2) | 12 (7.0) |  |
|  | Once or twice a week | 521 (7.3) | 10 (5.8) |  |
|  | 3-4 times a week | 267 (3.7) | 7 (4.1) |  |
|  | Almost every day | 667 (9.3) | 13 (7.6) |  |
| PLT (mean (SD)) |  | 213.3 (67.4) | 202.2 (68.6) | 0.032 |

Note: For continuous variables, the numbers in parentheses represent SD; for categorical variables, the numbers in parentheses represent percentage.
